# Nicotinamide Riboside Ameliorates Fructose-Induced Lipid Metabolism Disorders in Mice by Activating Browning of WAT, and May Be Also Related to the Regulation of Gut Microbiota

**DOI:** 10.3390/nu16223920

**Published:** 2024-11-17

**Authors:** Huaqi Zhang, Xuenuo Zhao, Li Zhang, Dan Sun, Yanzhen Ma, Yixian Bai, Xue Bai, Xi Liang, Hui Liang

**Affiliations:** Department of Nutrition and Food Hygiene, School of Public Health, Qingdao University, 308 Ningxia Road, Qingdao 266071, China; huaqi_erin@163.com (H.Z.); nuo0109996@163.com (X.Z.); zhangli200513@163.com (L.Z.); sundan202409@163.com (D.S.); mayanzhen97@163.com (Y.M.); bai_yixian@163.com (Y.B.); snowxue216@126.com (X.B.); liangxi6029@163.com (X.L.)

**Keywords:** nicotinamide riboside, fructose, lipid metabolism, browning, gut microbiota

## Abstract

Objectives: This study aims to observe the preventive effect of nicotinamide riboside (NR) on fructose-induced lipid metabolism disorders and explore its mechanism. Methods: Male C57BL/6J mice were fed a 20% fructose solution and given 400 mg/kg NR daily by gavage for 10 weeks. Results: The results indicated that NR supplementation significantly reduced the body weight, liver weight, white adipose tissue (WAT) weight, serum, and hepatic lipid levels. NR upregulated the protein expression levels of sirtuin-1 (SIRT1), AMP-activated protein kinase (AMPK), PR domain containing 16 (PRDM16), uncoupling protein 1 (UCP1), peroxisome proliferator-activated receptor-gamma coactiva-tor-1-alpha (PGC-1α), nuclear respiratory factor 1-encoding gene (NRF1), mitochondrial transcription factor A (TFAM), cluster of differentiation 137 (CD137), transmembrane protein 26 (TMEM26), and T-box 1 (TBX1). Moreover, NR enhanced the *Actinobacteria* and *Enterorhabdus* abundance. Spearman’s correlation analysis revealed that significant correlations exist between *Firmicutes, Bacteroidetes,* and *Erysipelotrichaceae* with browning-related indicators. Conclusions: In conclusion, NR could alleviate lipid metabolic abnormalities induced by fructose through activating SIRT1/AMPK-mediated browning of WAT. The mechanism by which NR improves fructose-induced lipid metabolism disorders may also be associated with the modulation of intestinal flora.

## 1. Introduction

Fructose, a natural monosaccharide, is primarily found in fruits and certain vegetables. Fructose is extensively used as a sugar substitute in food processing due to its high sweetness, low cost, and easy availability [1]. Epidemiological studies have confirmed that consumption of dietary fructose can cause dyslipidemia and increased hepatic lipogenesis [2,3]. In addition, research in vivo and in vitro has further revealed underlying mechanisms of lipid metabolism disorders induced by fructose exposure, such as induction of oxidative stress, islet inflammation [4], and fibroblast growth factor 21 resistance [5].

Recent studies have found fructose can affect the browning of white adipose tissue (WAT), which is another mechanism of fructose-induced lipid metabolism disorders [6]. The adipose tissues are generally classified into WAT and brown adipose tissue (BAT). WAT serves as an energy reserve, whereas BAT burns excess glucose and lipids, dissipating the energy as heat, also known as non-shivering thermogenesis [7]. Beige adipocytes, found mainly in WAT, can respond to external stimuli and transform into functional brown adipocytes, which possess abundant mitochondria and express uncoupling protein 1 (UCP1), with a thermogenic capacity similar to classical brown adipocytes [8]. In WAT, the process in which beige adipocytes express BAT-enriched genes is called the browning of WAT. Numerous studies have consistently confirmed that enhancing browning can effectively inhibit diet-induced obesity and ameliorate metabolism disorders, highlighting its important role in lipid metabolism [9,10]. Moreover, gut microbiota is also closely related to browning. It is found that fermented Ougan juice may induce browning of WAT by affecting the composition of intestinal flora, thus relieving obesity and lipid accumulation [11]. Therefore, better searching for activators of browning is of great significance for ameliorating lipid metabolism disorders caused by high-fructose exposure.

Nicotinamide riboside (NR) is a form of vitamin B_3_ that occurs naturally in milk and foods containing yeast [12]. NR as a precursor of nicotinamide adenine dinucleotide (NAD+) exerts many metabolic benefits by increasing NAD+ levels. Sirtuin-1 (SIRT1), an NAD+-dependent deacetylase, is a major regulator of the biological activity mediated by NR [13]. There is growing evidence that NR has the ability to ameliorate lipid metabolism. Moreover, NR can also modulate the composition and structure of gut microbiota [5,14,15]. AMP-activated protein kinase (AMPK) is one of the key downstream target genes of SIRT1, which has been confirmed by numerous in vitro studies [16,17,18]. AMPK has been demonstrated to play a crucial role in regulating beige adipocytes [19], which is associated with the upregulation of browning-related factors PR domain containing 16 (PRDM16) and peroxisome proliferator-activated receptor-gamma coactivator-1-alpha (PGC-1α) [20]. Therefore, we speculate NR may serve as a novel and effective activator of browning through the SIRT1/AMPK pathway and gut microbiota, which will be the first discovery of a new mechanism explaining how NR improves lipid metabolic disorders caused by high fructose.

In this study, mice were fed a 20% fructose solution and given 400 mg/kg NR daily by gavage for 10 weeks to observe the preventive effect of NR on fructose-induced lipid metabolism disorders and explore its mechanism by browning and intestinal flora.

## 2. Materials and Methods

### 2.1. Reagents and Chemicals

NR was provided by Jusheng Technology Co., Ltd. (Hubei, China). Primary antibodies used for western blotting include SIRT1, AMPK, PRDM16, UCP1, PGC-1α, nuclear respiratory factor 1-encoding gene (NRF1), cluster of differentiation 137 (CD137), transmembrane protein 26 (TMEM26), T-box 1 (TBX1) (Abcam, Cambridge, UK), mitochondrial transcription factor A (TFAM), (Affinity Biosciences, Cincinnati, OH, USA), ZO-1, claudin1 and occludin (Cell Signaling Technology, Danvers, MA, USA). β-actin was supplied by Abways (Shanghai, China). The corresponding secondary antibodies were obtained from Amersham (Bucks, UK).

### 2.2. Animals and Experimental Design

Male C57BL/6J mice (3 weeks, 12 ± 2 g) were purchased from Vital River (Beijing, China). The mice were kept in a room with a constant temperature of 20–25 °C, 50–55% relative humidity, and a 12 h light/dark cycle, with unrestricted access to food and water. After acclimated for 1 week, mice were randomly separated into a control group (CTRL), fructose group (FRU), and NR group (FRU+NR). Each group consisted of 10 mice. The CTRL group received tap water, and the other groups were given 20% (*w*/*v*) fructose solution. Mice in the CTRL and FRU groups were intragastrically administered with equal amounts of normal saline (0.1 mL/10 g), while mice in the FRU group was given a daily gavage of 400 mg/kg NR. Body weight was measured weekly. This study’s animals and protocols received approval from the Ethics Committee of the Medical College of Qingdao University (Approval number: QDU-AEC-2023362). After 10 weeks of intervention, all animals fasted for 12 h and were anesthetized with sodium pentobarbital for blood collection and then euthanized. Collective blood samples were centrifuged at 3000 rpm for 15 min at 4 °C. The liver and WAT were immediately gathered and weighed. The serum and tissue samples were stored at −80 °C until analysis. Cecal contents were collected into a dry, sterile tube and stored at −80 °C for microbiota analysis.

### 2.3. Serum Biochemical Analysis

The serum triglyceride (TG), total cholesterol (TC), low-density lipoprotein-cholesterol (LDL-C), and high-density lipoprotein-cholesterol (HDL-C) levels were detected with an automatic biochemical analyzer. Serum leptin and adiponectin levels were measured using enzymatic assay kits (R&D Systems, Minneapolis, MN, USA).

### 2.4. Liver Biochemical Analysis

Liver samples were homogenized with normal saline (1:9, g:mL) and centrifuged (2500 rpm and 15 min). The resulting supernatant was collected for measuring hepatic TG and non-esterified fatty acids (NEFA) levels using enzymatic assay kits (Jiancheng Bioengineering Institute, Nanjing, China).

### 2.5. Histological Analysis

The WATs were fixed in 10% formalin for two days, washed, dehydrated, and then embedded in paraffin. Five micrometer sections were prepared and stained with hematoxylin and eosin (H&E) for observation under optical microscopy (Olympus, Tokyo, Japan). Adipocyte size was quantified with ImageJ software (version 1.8.0) and expressed in average cross-sectional area (µm^2^) per adipocyte.

### 2.6. Immunohistochemical

Adipose tissue paraffin sections (5 µm) were dewaxed with xylene and treated with 3% H_2_O_2_. Next, the sections were incubated with the UCP1 primary antibody at 4 °C, washed in Tris-buffered saline (TBS), and then incubated with the corresponding secondary antibody at 37 °C for 30 min. Finally, the sections were followed by incubation with diaminobenzidine (DAB), counterstaining with hematoxylin, and observing under light microscopy.

### 2.7. Western Blotting

The methods of protein isolation, immunoblotting, and detection were followed in our previous research [21,22]. The protein expression levels of SIRT1, AMPK, PRDM16, UCP1, PGC-1α, NRF1, TFAM, CD137, TMEM26, TBX1, ZO-1, claudin1, and occludin were determined. β-Actin served as an internal control.

### 2.8. DNA Extraction and 16S rRNA Sequencing

Five cecal contents were selected from each group for 16S rRNA gene sequencing. As previously described [23], briefly, DNA was extracted from samples using a DNA extraction kit. Total DNA and integrity were examined. The V3–V4 regions of the 16S rRNA gene were amplified with the general primers (338F: 5′-ACTCCTACGGGAGGCAGCA3′ and 806R: 5′-GGACTACHVGGGTWTCTAAT-3′) and purified its products. Then, the pooled PCR products were sequenced (Illumina NovaSeq 6000) (San Diego, CA, USA). Quality checking and filtering of raw data using Trimmomatic. In order to determine the representative taxa for each group, the linear discriminant analysis (LDA) effect size (LEfSe) algorithm was applied with an LDA score threshold of 4.0. 

### 2.9. Statistical Analysis

Results are expressed as means ± standard deviation (SD). SPSS 23.0 (SPSS, Chicago, IL, USA) was used for one-way analysis of variance (ANOVA) to examine differences among multiple groups. Fisher’s LSD was performed when ANOVA had a significant result. The relationship between gut microbiota and browning-related indices and metabolic parameters was analyzed by Spearman’s correlation test. *p* < 0.05 was considered statistically significant.

## 3. Results

### 3.1. Effect of NR on Body Weight and Liver Weight

**Figure 1:**
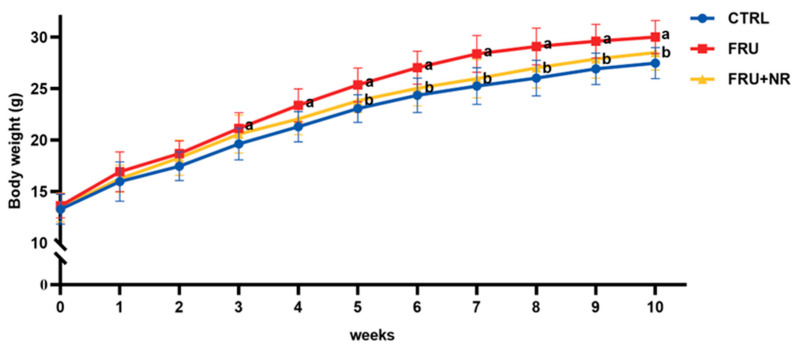
Effect of NR on body weight. Values are represented as mean ± SD (*n* = 10). ^a^ *p* < 0.05 vs. CTRL group; ^b^ *p* < 0.05 vs. FRU group.

Mice in the FRU group exhibited a higher body weight than in the CTRL group starting from week 3. NR supplementation had alleviated excessive weight gain due to high fructose (*p* < 0.05; Figure 1). As demonstrated in Table 1, at the end of 10 weeks, the FRU+NR group had lower body weight gain than the FRU group (*p* < 0.05). The energy intake was higher in the FRU group than in the CTRL group (*p* < 0.05), but no significant differences in energy intake were observed between the FRU and FRU+NR groups. Furthermore, liver weight and liver index in the FRU group were higher than in the CTRL group (*p* < 0.05), and liver weight and liver index were noticeably lower in the FRU+NR group than in the FRU group (*p* < 0.05). 

### 3.2. Effect of NR on Serum and Hepatic Lipids Levels

As shown in Table 2, the serum TG and TC levels were remarkably higher in the FRU group than in the CTRL group (*p* < 0.05). The FRU+NR group had remarkably lower serum TG and TC levels than the FRU group (*p* < 0.05). No significant differences were observed in serum LDL-C and HDL-C levels among the three groups (*p* > 0.05). Compared with the CTRL group, the serum leptin was elevated in the FRU group, while the serum adiponectin was markedly decreased (*p* < 0.05). In contrast, compared to the FRU group, the serum leptin was reduced, while the serum adiponectin was noticeably elevated in the FRU+NR group (*p* < 0.05). Hepatic TG and NEFA levels in the FRU group were higher than those in the CTRL group (*p* < 0.05), whereas the FRU+NR group exhibited dramatically lower hepatic TG and NEFA levels than the FRU group (*p* < 0.05).

### 3.3. Effect of NR on Morphology and Structure in White Adipose Tissues

**Figure 2:**
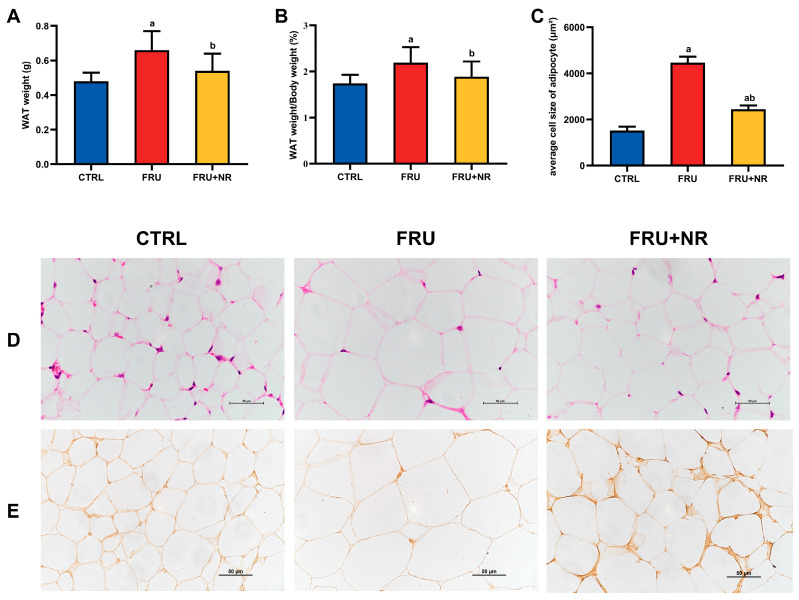
Effect of NR on morphology and structure in white adipose tissues. (**A**) WAT weight; (**B**) WAT/body weight ratio; (**C**) average cell size of WAT adipocyte; (**D**) representative H&E staining of WAT (40×, scale bars = 50 µm); (**E**) immunohistochemical images of UCP1. Values are represented as mean ± SD. ^a^ *p* < 0.05 vs. CTRL group; ^b^ *p* < 0.05 vs. FRU group.

As demonstrated in Figure 2, the WAT weight and WAT weight/body weight were dramatically higher in the FRU group than those in the CTRL group, while these parameters were noticeably lower in the FRU+NR group relative to the FRU group (*p* < 0.05; Figure 2A,B). H&E staining exhibited that adipocyte size in the FRU group was larger than in the CTRL group (*p* < 0.01; Figure 2C), which was consistent with the weight changes in WATs. Supplementation of NR showed smaller adipocytes and presented more intense staining (confirmed by the immunohistochemical results (Figure 2E)) of the WAT. 

### 3.4. Effect of NR on the Expression of Proteins Related to Lipid Metabolism

**Figure 3:**
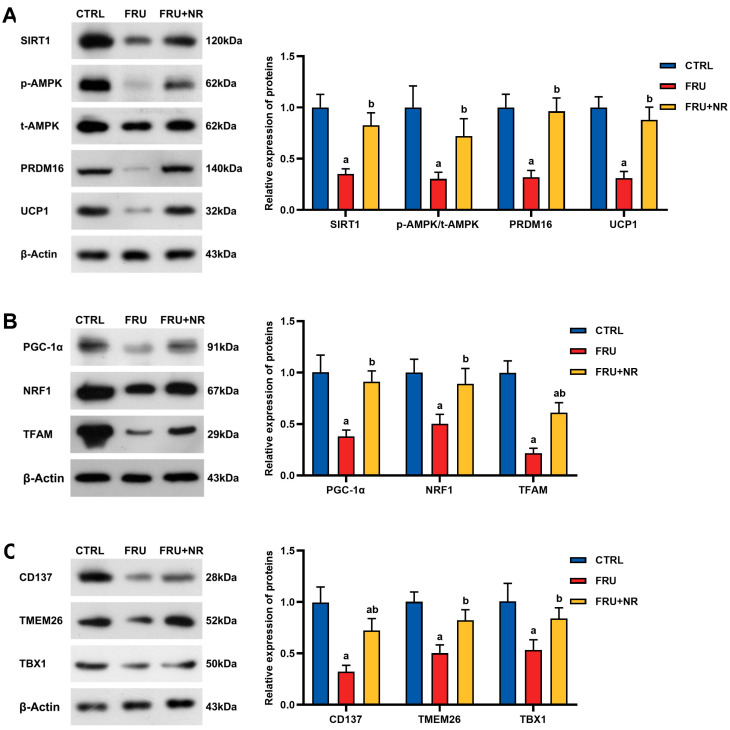
Effects of NR on the lipid metabolism-related proteins expression of adipose tissues. (**A**) SIRT1, p-AMPK, t-AMPK, PRDM16, and UCP1 expressions; (**B**) PGC-1α, TFAM, and NRF1 expressions; (**C**) CD137, TMEM26, and TBX1 expressions. Values are represented as mean ± SD (*n* = 3). ^a^ *p* < 0.05 vs. CTRL group; ^b^ *p* < 0.05 vs. FRU group.

In contrast to the CTRL group, SIRT1, p-AMPK/t-AMPK, PRDM16 and UCP1 expressions were dramatically lower in the FRU group. SIRT1, p-AMPK/t-AMPK, PRDM16, and UCP1 levels were remarkably higher in the FRU+NR group than in the FRU group (*p* < 0.05; Figure 3A). The level of proteins related to mitochondrial biogenesis are shown in Figure 3B. The FRU group exhibited dramatically lower PGC-1α, NRF1, and TFAM ex-pressions when relative to the CTRL group, while expressions of these proteins in the FRU+NR group were remarkably restored (*p* < 0.01). Beige adipocyte marker expressions were demonstrated in Figure 3C. CD137, TMEM26, and TBX1 levels in the FRU group were dramatically lower than in the CTRL group, while they were dramatically recovered in the FRU+NR group (*p* < 0.05).

### 3.5. Effect of NR on Tight Junction Protein Expression

**Figure 4:**
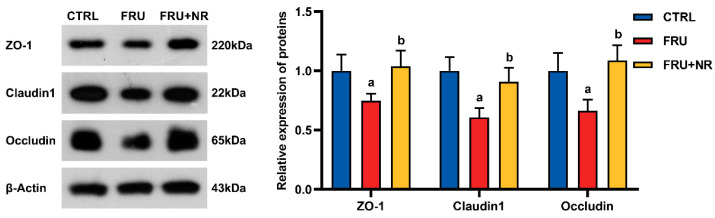
Effects of NR on ZO-1, claudin1 and occludin expressions. Values are represented as mean ± SD (*n* = 3). ^a^ *p* < 0.05 vs. CTRL group; ^b^ *p* < 0.05 vs. FRU group.

As demonstrated in Figure 4, ZO-1, claudin1, and occludin expressions in the FRU group were noticeably lower than in the CTRL group. These proteins were expressed higher in the FRU+NR group than in the FRU group (*p* < 0.05).

### 3.6. Effect of NR on Gut Microbiota

**Figure 5:**
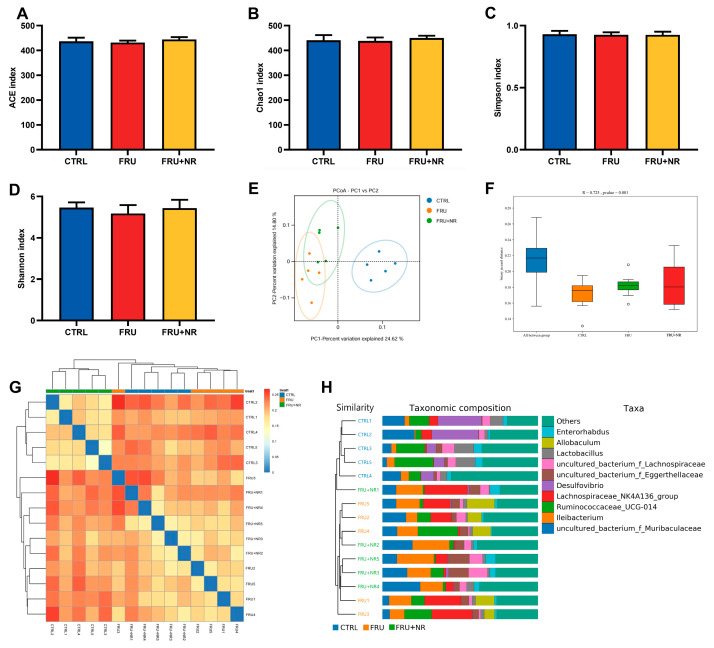
Effects of NR on α-diversity and β-diversity of gut microbiota. (**A**) ACE index; (**B**) Chao1 index; (**C**) Simpson index; (**D**) Shannon index; (**E**) PCoA; (**F**) ANOSIM analysis; (**G**) heatmap; (**H**) UPGMA. Values are represented as mean ± SD (*n* = 5).

To analyze the impact of FRU-exposed and NR-treated on richness (ACE index and Chao 1 index) and diversity (Shannon index and Simpson index) of gut microbiota, the changes in alpha diversity were investigated (Figure 5A–D). Although the Shannon index of the FRU group had a significant downward trend, there were no statistically significant differences among all groups.

Using beta-diversity analysis based on the binary Jaccard algorithm, the principal coordinate analysis (PCoA) showed that the microbial communities were different among groups; the clusters in the CTRL group were obviously separated from those in the FRU group, whereas the clusters of the FRU+NR group and the CTRL group were relatively close (Figure 5E). Moreover, the analysis of similarities (ANOSIM) revealed the differences between the groups were greater than those within the groups (R = 0.725, *p* = 0.001; Figure 5F). The heatmaps and sample hierarchical clustering tree confirmed these results; compared with the FRU group, the similarity between the FRU+NR and CTRL samples was greater (Figure 5G,H). 

**Figure 6:**
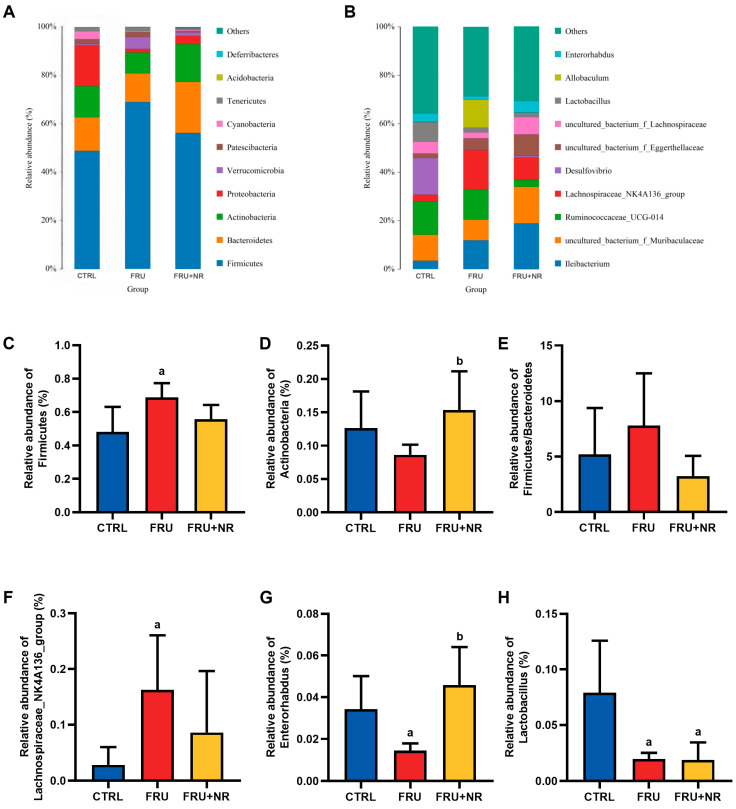
Effects of NR on gut microbiota composition in each group. (**A**) Phylum; (**B**) genus; (**C**) *Firmicutes* abundance; (**D**) *Actinobacteria* abundance; (**E**) F/B ratio; (**F**) *Lachnospiraceae_NK4A136_group* abundance; (**G**) *Enterorhabdus* abundance; (**H**) *Lactobacillus* abundance. ^a^ *p* < 0.05 vs. CTRL group; ^b^ *p* < 0.05 vs. FRU group.

Taxonomic annotation showed that the gut microbiota was predominantly made up of Firmicutes, Bacteroidetes, Actinobacteria, and Proteobacteria at the phylum level (Figure 6A). Firmicutes abundance was remarkably higher in the FRU group when relative to the CTRL group (*p* < 0.05; Figure 6C). Actinobacteria abundance displayed a marked increase in the FRU+NR group when relative to the FRU group (*p* < 0.05; Figure 6D). At the genus level, the top ten genera are presented in Figure 6B. Lachnospiraceae_NK4A136_group abundance in the FRU group was dramatically higher relative to the CTRL group (*p* < 0.05; Figure 6F). Enterorhabdus and Lactobacillus abundance in the FRU group were remarkably lower than in the CTRL group, whereas NR supplementation had markedly elevated the Enterorhabdus abundance. (*p* < 0.05; Figure 6G,H). 

**Figure 7:**
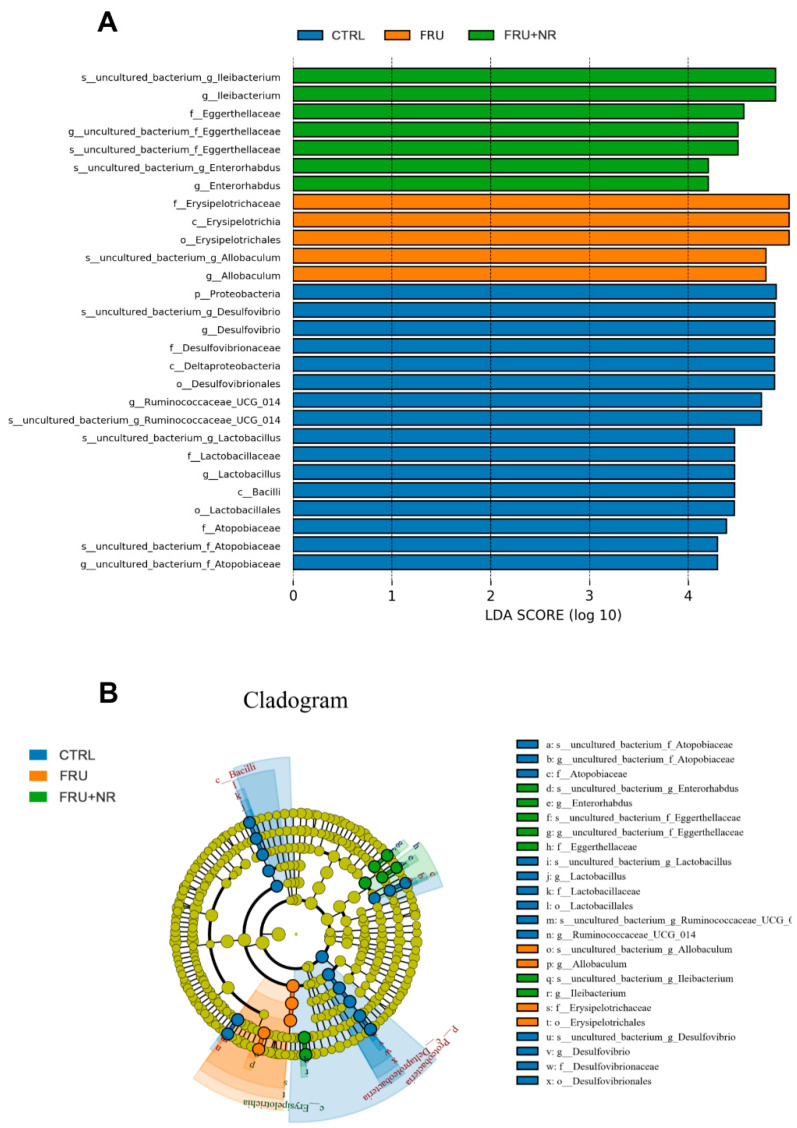
LEfSe analysis. (**A**,**B**) LEfSe analysis was used to identify the total bacteria (*p* < 0.05 and LDA score > 4).

LEfSe analysis could find the biomarker with a statistical difference between different groups. As illustrated in Figure 7, the results revealed that there were significant differences among 28 OTUs at the level of phylum (1 OTU), class (3 OTUs), order (3 OTUs), family (5 OTUs), genus (8 OTUs), and species (8 OTUs). Among the significantly different OTUs, the highest abundance of bacteria in the CTRL group included *Proteobacteria* and *Desulfovibrio*; *Erysipelotrichaceae* was the highest abundance of bacterium in the FRU group, while *Ileibacterium* was the highest abundance of bacterium in the FRU+NR group.

**Figure 8:**
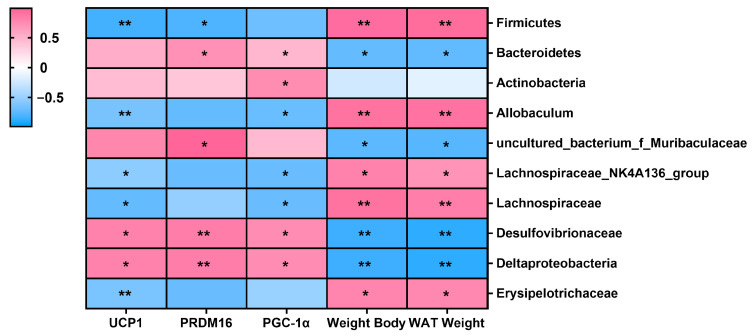
Spearman correlation heatmap between the gut microbiota and browning-related indicators and metabolic parameters among the CTRL, FRU, and FRU+NR groups (* *p* < 0.05, ** *p* < 0.01).

The relationship between bacterial taxa and browning-related indicators and metabolic parameters is shown in Figure 8. *Bacteroidetes, Actinobacteria*, *uncultured_bacterium_f_Muribaculaceae,* and *Deltaproteobacteria* were positively correlated with browning-related indicators, especially PRDM16 and PGC-1α (*p* < 0.05). *Bacteroidetes* and *uncultured_bacterium_f_Muribaculaceae* exhibited an inverse correlation with body weight and WAT weight (*p* < 0.05). *Firmicutes, Allobaculum Lachnospiraceae_NK4A136_group,* and *Erysipelotrichaceae* exhibited an inverse correlation with UCP1, PRDM16, and PGC-1α proteins (*p* < 0.05), while exhibiting a positive association with metabolic parameters (*p* < 0.05).

## 4. Discussion

This study is the first to show that NR can alleviate lipid metabolism disorders by activating the browning of WAT in fructose-fed mice. NR can increase UCP1 expression and mitochondrial biogenesis through activating the SIRT1/AMPK pathway, thereby inducing browning. Moreover, NR has the effect of regulating the gut microbial composition, which may be involved in the occurrence of browning and improve lipid metabolism disorders.

The excessive consumption of added sugar has become a major public health problem, especially as fructose intake has substantially increased in Western diets over the past few decades [24]. In humans, long-term consumption of high fructose can cause lipid metabolism disorders, leading to hypertriglyceridemia, obesity, and insulin resistance [25,26]. Experimental feeding studies have confirmed that high-fructose exposure (13% or 66%) can lead to lipid metabolism disorders by inducing de novo lipogenesis and decreasing β-oxidation and increase body weight [27,28]. In this study, we intervened mice with a 20% fructose solution and found that the fructose diet had increased body weight and elevated serum and liver lipid levels, which is consistent with previous findings. 

Some phytochemicals have been found to improve lipid metabolism disorders caused by high fructose exposure. Water extracts of shepherd’s purse show potential in improving lipid metabolism disorders and hepatic steatosis in fructose-fed mice, which effect is achieved through the modulation of the FAS/ACC pathway [29]. Similarly, the loquat fruit extract has been shown to contribute to lipid homeostasis in mice fed a fructose diet by inhibiting hepatic lipogenesis [30]. NR is a form of vitamin B_3_ that is naturally found in yeast, bacteria, and mammals [12]. NR can reverse alcohol-induced liver injuries by regulating lipid metabolism disorders [14,31]. NR also can enhance oxidative metabolism in obese mice induced by high fat, thereby improving metabolic abnormalities [32]. Therefore, the prospect of NR to prevent lipid metabolism disorders caused by high-fructose exposure attracted our attention. In previous studies [5,14], 400 mg kg^−1^ NR has been utilized extensively, and numerous beneficial physiological impacts of NR have been explored at this dose. In this study, we administered fructose exposure at the same time as giving a daily dose of 400 mg kg^−1^ NR and found that body weight, liver weight, serum TG level, and hepatic TG and NEFA levels were diminished in the FRU+NR group compared with the FRU group. These results indicated that NR could prevent lipid disorders caused by a fructose diet to some extent. In our previous study, similar results were observed, and we had firstly found [5] that NR might decrease hepatic lipogenesis and promote lipolysis by improving FGF21 resistance. However, FGF21 resistance is not enough to fully explain the regulatory influence of NR on lipid metabolism, and there may be more new mechanisms worth exploring. Therefore, other potential mechanisms by which NR regulated lipid metabolism were explored in this study.

As the central controller of lipid homeostasis, adipose tissue serves a vital function in human energy metabolism and endocrine, and its thermogenic role in lipid metabolism has been widely explored recently. In general, adipocytes have been divided into white adipocytes, beige adipocytes, and brown adipocytes. White adipocytes are defined by a prominent lipid droplet and a limited number of mitochondria, serving both energy storage and endocrine functions. In contrast, brown adipocytes contain multiple lipid droplets and are more mitochondria-rich, functioning primarily to dissipate energy as heat [33]. Beige adipocytes are an inducible form of thermogenic adipocytes in WAT depots, which are different from the classical brown adipocyte source. When subjected to external stimuli, beige adipocytes become functional brown adipocytes, generating heat and increasing energy expenditure [34]. This phenomenon in which brown-like adipocytes can be detected in WAT and the expression of brown adipose marker genes is increased is known as the browning of WAT. It is worth noting that the fructose diet had been found to cause adipocyte hypertrophy and impair brown adipocyte thermogenesis [35]. It has been reported that NR supplementation in the early postnatal period has a sex-dependent long-term effect on the thermogenesis of adipose tissues in mice [36]. However, whether NR can prevent lipid metabolism disorders by inducing browning under adverse conditions of a fructose diet has not been reported. 

In this study, it was found that the white adipocytes were significantly reduced and multilocular brown-like cells appeared in the FRU+NR group. UCP1 is the hallmark of browning. The ability of brown adipocytes to anti-obesity and fulfill their thermogenic function relies on the presence of UCP1 protein [37]. CD137, TMEM26, and TBX1 are beige adipocyte markers. Genes expressed in a beige-selective manner include TBX1, which can distinguish beige adipocytes from both brown adipocytes and from white adipocytes from the WAT depot. Primary beige adipocytes expressed CD137 and TMEM26 on the cell surface, and only those adipocytes that differentiate from beige preadipocytes are capable of activating the thermogenic gene program [34,38]. One study showed that high levels of CD137, TMEM26, and TBX1 were accompanied by elevated UCP1 protein [10]. Therefore, to further confirm whether NR has the potential for activating browning, beige adipocyte marker protein and the core protein of browning were detected. As expected, compared with the FRU group, UCP1 expression and the characteristic protein levels of beige adipocytes (CD137, TMEM26, TBX1) were increased in the FRU+NR group. Several studies have reported the influence of leptin and adiponectin on browning. Expression of UCP1 is impaired in leptin-resistant obese mice; conversely, the enhanced levels of adiponectin comply with the enhanced thermogenesis in beige adipocytes [39,40]. In this study, we also observed a decrease in serum leptin and an increase in adiponectin in the FRU+NR group compared to the FRU group. The above results of pathology, marker protein expression, and adipokine levels indicated that NR had the ability to induce browning of WAT.

NR is a natural NAD+ precursor that can be metabolized into NAD+ through the Nrks pathway [41]. Supplementing with NR can offer metabolic benefits through the elevation of NAD+ levels. In addition to various forms of vitamin B_3_ and tryptophan, NAD+ is also affected by nutritional status; for example, NAD+ biosynthesis can be inhibited by overnutrition or obesity [42,43]. In our previous study [5], high-fructose exposure was found to decrease NAD+ levels, while NR supplementation significantly increased NAD+ levels. NAD+ is a cofactor involved in cellular redox and energy metabolism, serving as a substrate for various key metabolic enzymes [32]. SIRT1 is an NAD+-dependent histone deacetylase, whose activity can be regulated by the NAD+ levels [43]. Therefore, the metabolic benefits provided by NR can be realized by modulating SIRT1 action with NAD+ as a coenzyme. For example, NR can enhance endothelial precursor cell function by mediating the SIRT1/AMPK pathway [18] and can also alleviate inflammation and oxidative stress through activating SIRT1 in macrophages [13]. The AMPK is an important intracellular energy stress sensor, which can be phosphorylated by SIRT1 through LKB1 deacetylation [44]. SIRT1 knockdown has been reported to reduce the phosphorylation level of AMPK [45]. Studies with both in vivo and in vitro evidence have confirmed that phosphorylated AMPK is instrumental in activating the browning of WAT, maintaining BAT, and promoting adaptive thermogenesis [19,20]. As expected, this study found that NR supplementation elevated the SIRT1 expression and phosphorylated AMPK in WAT of high-fructose mice, suggesting that the browning of WAT regulated by NR may be mediated by the SIRT1/AMPK pathway. 

PRDM16 is an indispensable transcriptional regulator for brown adipogenesis, which is capable of maintaining the beige phenotype of adipocytes and regulating the expression program of browning-related genes and thermogenesis [46,47]. There is evidence confirming that AMPK activation causes an increase in α-KG levels and demethylation of DNA on the PRDM16 promoter, thereby promoting the expression of PRDM16 [47]. PRDM16 can directly bind to the promoter region of UCP1 to upregulate its transcription and thus participate in the synthesis and thermogenesis of UCP1 in brown adipocytes [48]. In this study, we observed that with the upregulation of SIRT1 and AMPK expression levels, the PRDM16 expression was elevated in the FRU+NR group compared with the FRU group, which may explain the high expression of UCP1. The results indicated that NR can induce the acquisition of BAT features in white adipocytes through the SIRT1/AMPK/PRDM16 signaling pathway. 

In addition, the thermogenic capacity is also influenced by the mitochondrial abundance. UCP1 is situated in the inner mitochondrial membrane, where there is a protein proton channel. Upon exposure to cold or external stimuli, this channel opens to uncoupling of oxidative phosphorylation, and UCP1 serves as the key protein for this uncoupling process, enabling the dissipation of energy as heat [46]. Consequently, the facilitation of mitochondrial biogenesis is equally indispensable for the browning. PGC-1α serves as a key transcriptional regulator of mitochondrial biogenesis, with NRF1 serving as its primary target. Activation of NRF1 results in elevated TFAM expression, which predominantly facilitates the replication and maintenance of mitochondrial DNA [37,44]. Extensive investigation has demonstrated that AMPK can enhance PGC-1α level; the AMPK/PGC-1α signaling pathway contributes to the enhancement of mitochondrial function in db/db mice [49]. In in vitro experiments, AMPK was found to directly activate PGC-1α protein at threonine-177 and serine-538 by a direct phosphorylation [50]. In this research, the expressions of PGC-1α and its downstream protein NRF1 and TFAM were significantly higher in FRU+NR group compared to FRU group, suggesting that NR supplementation not only upregulates the PRDM16 and UCP1 expression, but also promote mitochondrial biogenesis, thereby inducing non-shivering thermogenesis, all of which are SIRT1/AMPK dependent effect.

In emerging research, the relationship between gut microbiota and lipid metabolism has been extensively described, and it has been suggested that dysregulation of gut microbiota is related to various diseases, including obesity, hyperlipidemia [51], and insulin resistance [52]. Recently, emerging evidence has revealed that specific bacteria may be involved in the browning of WAT [53]. Short-chain fatty acids have been reported to act as substrates for UCP1, facilitating UCP1-mediated heat generation. *Enterococcus faecalis* drove Myristoleic acid production via encoding ACOT gene, thereby increasing BAT activity and beige adipocytes formation [54]. Previous studies have provided substantial evidence linking fructose to disturbances in intestinal microecology, including reduced microbiota diversity and compromised gut barrier integrity [55]. However, whether NR can regulate the disturbance of microbiota induced by fructose exposure and whether the microbiota can further induce browning is still unknown.

In this study, sequencing analysis was conducted on the microbiomes from three groups. The diversity analysis revealed that the similarity of composition and structure of gut microbiota between the FRU+NR group and CTRL group was greater than those between the FRU group and CTRL group. At the phylum level, we found that a fructose diet led to an increase in *Firmicutes* abundance. *Firmicutes*, the most abundant phylum in gut microbiota, are accountable for energy resorption and obesity [56]. It had been reported that *Firmicutes* was significantly increased in obese children and a positive correlation between *Firmicutes* and BMI [57]. Additionally, animal experiments had found the body mass index and blood lipid in high-fat rats were positively associated with the *Firmicutes* [30]. *Actinobacteria* offer numerous potential benefits for humans, serving as sources of novel antibiotics, anticancer agents, and other secondary metabolites [58]. This study found the enrichment of *Actinobacteria* in NR-supplemented mice. In a previous study, gut microbiota analysis revealed that *Salvia miltiorrhiza* extract could promote the *Actinobacteria* and *Proteobacteria* abundance while decreasing the growth of *Firmicutes*, thereby reversing dysbacteriosis induced by high fat [59]. At the genus level, *Lachnospiraceae_NK4A136_group* is generally considered harmful; *Lachnospiraceae* has been linked to several chronic diseases, including diabetes and depression [57]. High fat and high fructose induced changes in various microflora in mice, including the elevation of *Lachnospiraceae_NK4A136_group* [60]. Comparable findings were noted in this study; *Lachnospiraceae_NK4A136_group* abundance in the FRU group was higher than in the CTRL group, but there was no significant difference between the two groups after NR intervention. *Enterorhabdus* and *Lactobacillus* are known as beneficial bacteria. *Enterorhabdus* is a butyric acid-producing bacterium in the gastrointestinal tract [61]. Short-chain fatty acids of microbial origin are involved in regulating lipid metabolism in both intestinal and peripheral tissues. Probiotic supplementation, including specific butyric acid-producing bacteria, demonstrated anti-obesity effects in both animal and population studies [62]. *Lactobacillus* acts as a probiotic and can be used to lower cholesterol in dairy products [63]. *Laminaria japonica* fermented with *Lactobacillus* alleviated abnormal lipid metabolism in high-fat-diet rats by regulating intestinal flora and liver gene profiles, which might be served as a functional food for preventing hyperlipidemia [51]. In this study, the abundance of *Enterorhabdus* and *Lactobacillus* was dramatically decreased in the FRU group compared to the CTRL group, while NR supplementation dramatically increased the abundance of *Enterorhabdus*. However, no increase in *Lactobacillus* was observed in the FRU+NR group, suggesting that NR might not be exerting benefits with *Lactobacillus* as the primary target. In order to understand the specific bacterial taxa of each group, LEfSe analysis was conducted. The findings revealed that the abundance of *Erysipelotrichaceae* was highest in the FRU group. Recently, several investigations have demonstrated that the number of certain *Erysipelotrichaceae* strains is significantly increased in patients with obesity and hepatic steatosis [64]. *Ileibacterium* abundance in the FRU+NR group was noticeably higher than in other groups. A previous study demonstrated that hulless barley improved fat accumulation in liver tissue and intestinal flora dysregulation induced by high fat, promoting some beneficial bacteria such as *Lactobacillus*, *Bifidobacterium*, and *Ileibacterium* [65]. Another study pointed out that *Ileibacterium* significantly enriched by Xie Zhuo Tiao Zhi decoction was negatively correlated with biomarkers of liver injury [66]. 

To further determine the relationship between gut microbiota and browning, Spearman correlation analysis was conducted to assess the association between the relative abundance of gut microbiota and metabolic parameters and browning-related indicators. *Firmicutes*, *Allobaculum*, *Lachnospiraceae,* and *Erysipelotrichaceae* showed a remarkably positive relationship with body weight and WAT weight. In previous studies, *Allobaculum*, family *Lachnospiraceae,* and *Erysipelotrichaceae* were also found to be positively correlated with lipid metabolic parameters (TG, TC, body weight) [67,68]. *Bacteroidetes* abundance was positively associated with PRDM16, while it was inversely correlated with body weight and WAT weight. Studies have shown that intragastric injection of *Bacteroidetes* spp. can inhibit weight gain and induce the expression of UCP1 [69]. Furthermore, in a previous study, *Actinobacteria* had a positive correlation with browning-related indicators of retroperitoneal WAT (PGC-1α and PRDM16), and class *Deltaproteobacteria* and family *Desulfovibrionaceae* (within *Proteobacteria*) were significantly positively correlated with UCP1 in retroperitoneal WAT [67]. Consistent with these studies, we found that *Actinobacteria*, *Deltaproteobacteria,* and *Desulfovibrionaceae* were positively associated with browning-related indicators. These results suggested that NR-induced changes in specific bacteria could ameliorate lipid metabolism abnormalities and may play their role by affecting browning. Based on the results, we speculated that the gut microbiota could be a contributing factor in the promotion of browning by NR, but the mechanisms behind these results still require further investigation.

## 5. Conclusions

This study found that NR can ameliorate the increase in body weight, serum, and hepatic TG levels caused by fructose exposure and firstly found that it may be related to the browning of WAT mediated by the SIRT1/AMPK signaling pathway and its downstream proteins PRDM16 and PGC-1α. Moreover, NR could regulate the disturbance of intestinal flora under fructose exposure. The correlation between gut microbiota and browning suggests that the change in microbiota may also be one of the possible mechanisms of inducing browning by NR. As a limitation of this study, it did not provide direct evidence that the gut microbiome contributes to browning. In future studies, the modulation of intestinal flora on browning and its mechanism under NR intervention can be further definitively confirmed by fecal microbiota transplantation. NR supplementation may be a new strategy for preventing lipid metabolic abnormalities induced by fructose.

## Figures and Tables

**Table 1:** Effect of NR on body weight gain and liver weight.

Group *	CTRL	FRU	FRU+NR
Body weight gain (g)	14.21 ± 1.38	16.41 ± 1.38 ^a^	15.06 ± 1.47 ^b^
Energy intake (kcal/day)	9.83 ± 1.28	12.75 ± 1.29 ^a^	12.29 ± 1.45 ^a^
Liver weight (g)	0.98 ± 0.06	1.14 ± 0.08 ^a^	1.05 ± 0.09 ^b^
Liver index (%)	3.58 ± 0.11	3.80 ± 0.13 ^a^	3.67 ± 0.13 ^b^

* *n* = 10 per group. ^a^ *p* < 0.05 vs. CTRL group; ^b^ *p* < 0.05 vs. FRU group. Values are represented as mean ± SD.

**Table 2:** Effect of NR on serum and hepatic lipids levels.

Group *	CTRL	FRU	FRU+NR
Serum TG (mmol/L)	1.12 ± 0.15	1.39 ± 0.17 ^a^	1.16 ± 0.12 ^b^
Serum TC (mmol/L)	3.03 ± 0.26	3.32 ± 0.32 ^a^	3.06 ± 0.27 ^b^
Serum LDL-C (mmol/L)	1.63 ± 0.14	1.73 ± 0.15	1.58 ± 0.19
Serum HDL-C (mmol/L)	0.78 ± 0.13	0.87 ± 0.10	0.82 ± 0.09
Serum leptin (ng/ml)	2.07 ± 0.12	2.34 ± 0.17 ^a^	2.19 ± 0.20 ^b^
Serum adiponectin (ng/ml)	66.11 ± 7.02	50.83 ± 7.49 ^a^	59.54 ± 8.87 ^b^
Hepatic TG (mmol/g protein)	0.18 ± 0.03	0.22 ± 0.02 ^a^	0.19 ± 0.02 ^b^
Hepatic NEFA (mmol/g protein)	0.15 ± 0.02	0.18 ± 0.03 ^a^	0.16 ± 0.02 ^b^

* *n* = 10 per group. ^a^ *p* < 0.05 vs. CTRL group; ^b^ *p* < 0.05 vs. FRU group. Values are represented as mean ± SD. TG: triglyceride; TC: total cholesterol; LDL-C: low-density lipoprotein cholesterol; HDL-C: high-density lipoprotein cholesterol; NEFA: non-esterified fatty acids.

## Data Availability

The datasets used and/or analyzed during the current study are available from the corresponding author upon reasonable request. The data are not publicly available due to laboratory policies and confidentiality agreements.
